# Clinical and Epidemiologic Characteristics and Therapeutic Management of Patients with *Vibrio* Infections, Bay of Biscay, France, 2001–2019

**DOI:** 10.3201/eid2812.220748

**Published:** 2022-12

**Authors:** Florence Hoefler, Xavier Pouget-Abadie, Mariam Roncato-Saberan, Romain Lemarié, Eve-Marie Takoudju, François Raffi, Stéphane Corvec, Morgane Le Bras, Charles Cazanave, Philippe Lehours, Thomas Guimard, Caroline Allix-Béguec

**Affiliations:** Centre Hospitalier La Rochelle, La Rochelle, France (F. Hoefler, X. Pouget-Abadie, M. Roncato-Saberan, R. Lemarié, C. Allix-Béguec);; Centre Hospitalier Départemental Vendée, La Roche sur Yon, France (E.-M. Takoudju, T. Guimard);; Centre Hospitalier Universitaire de Nantes, Nantes, France (F. Raffi, S. Corvec, M. Le Bras);; Centre Hospitalier Universitaire de Bordeaux, Bordeaux, France (C. Cazanave, P. Lehours)

**Keywords:** *Vibrio* infections, diagnosis, therapeutics, bacteria, bacterial infections, antibacterial agents, amputation, death, Bay of Biscay, France, antimicrobial resistance

## Abstract

Noncholera vibriosis is a rare, opportunistic bacterial infection caused by *Vibrio* spp. other than *V. cholerae* O1/O139 and diagnosed mainly during the hot summer months in patients after seaside activities. Detailed knowledge of circulating pathogenic strains and heterogeneities in infection outcomes and disease dynamics may help in patient management. We conducted a multicenter case-series study documenting *Vibrio* infections in 67 patients from 8 hospitals in the Bay of Biscay, France, over a 19-year period. Infections were mainly caused by *V. alginolyticus* (34%), *V. parahaemolyticus* (30%), non-O1/O139 *V. cholerae* (15%), and *V. vulnificus* (10%). Drug-susceptibility testing revealed intermediate and resistant strains to penicillins and first-generation cephalosporins. The acute infections (e.g., those involving digestive disorder, cellulitis, osteitis, pneumonia, and endocarditis) led to a life-threatening event (septic shock), amputation, or death in 36% of patients. Physicians may need to add vibriosis to their list of infections to assess in patients with associated risk factors.

## Abstract 2

Acute vibriosis led to serious adverse events in more than one third of patients with underlying conditions.

Some opportunistic pathogens associated with marine environments are already known but until now have caused rare infectious diseases. Among those pathogens are *Vibrio* spp. other than the well-known *V. cholerae* belonging to serogroups O1 and O139, which causes cholera. *Vibrio* spp. are gram negative, curved, rod-shaped bacteria that are natural inhabitants of the aquatic environment (*1*). *Vibrio* infections can be very severe or even fatal; they cause gastroenteritis, severe bacterial cellulitis, or necrotizing fasciitis and can lead to septic shock. Infections are more common in patients with multiple underlying conditions, including liver disease, heart failure, diabetes, liver cirrhosis, alcohol abuse, and immunocompromising conditions (*2*–*5*). *Vibrio* spp. can also cause mild diseases, such as chronic ear infections, which are more likely to affect younger patients (*6*). Humans acquire *Vibrio* infections after eating contaminated raw seafood, especially oysters, or after exposing an injury to the marine environment (*7*). Infections occur mainly during the hot summer months, which is probably attributable to higher water temperatures (*8*,*9*) and to increased seawater-related activities. 

Because vibriosis is a relatively rare disease and is not reported in most national surveillance systems, the global incidence rate of *Vibrio* spp. infections other than *V. cholerae* O1/O139 is underestimated. In the United States, where those infections are notifiable, a marked seasonal distribution and an increasing incidence rate have been observed (*10*–*12*). Because of their rarity, *Vibrio* infections are very poorly known and therefore probably underdiagnosed. Delays in therapeutic management and, in particular, in the prescription of a targeted antibiotic regimen have been documented (*13*). Our study aimed to make an inventory of *Vibrio* infections diagnosed in hospitals in the Bay of Biscay on the west coast of France and to describe the clinical and epidemiologic characteristics of the patients and their therapeutic management.

## Methods

### Study Design and Participants

We conducted a multicenter case-series study based on data collected from 8 tertiary and secondary care hospitals in the Bay of Biscay, France. We included all cases of vibriosis other than those caused by *V. cholerae* O1/O139 diagnosed during January 2001–December 2019.

### Diagnosis and Susceptibility Test

We defined a *Vibrio* infection as a positive biologic sample (e.g., blood, skin sample, surgical biopsy, stool sample, bronchoalveolar lavage, and ear sample) to a *Vibrio* species other than *V. cholerae* O1/O139. Conventional microbiologic methods were used to isolate bacteria from the different types of samples. BACTEC automated blood culture system (Becton, Dickinson and Company, https://www.bd.com) was used before conventional culture for the rapid detection of microorganisms in blood samples. Since 2018, automated diagnostic testing of stool samples for direct qualitative detection and differentiation of enteric bacterial pathogens has been performed with the BD MAX Enteric Bacterial Panel performed on the BD MAX system (Becton, Dickinson and Company). Before 2014, API 20 E biochemical tests (bioMérieux, https://www.biomerieux.com) were used for species identification. Since 2014, those tests have been replaced by the use of the Bruker Biotyper matrix-assisted laser desorption/ionization time-of-flight mass spectrometry (https://www.bruker.com). Antibiotic susceptibility was tested on the main class of antibiotics, including penicillins, cephalosporins, carbapenems, and fluoroquinolones. Antimicrobial susceptibility testing was performed using Mueller–Hinten agar disk diffusion tests (AST Disks; Bio-Rad, https://www.bio-rad.com) in accordance with the recommendations of the Committee on Antimicrobial Susceptibility of the French Society for Microbiology. Most isolates were sent to the national reference center for confirmation of species identification and susceptibility results.

### Ethics and Regulation

This study received a favorable opinion from the Committee of Expertise for Health Research, Studies, and Evaluations (registration no. TPS 1170745). It was authorized by the Commission Nationale de l’Informatique et des Libertés (decision no. DR-2020–125). A letter explaining the study and the patients’ rights regarding the use of their data was sent to the last known address of the patients. This study was registered on clinicaltrials.gov (identifier NCT04451707).

### Variables and Statistical Methods

We retrieved sociodemographic data, sea-related activity, and clinical and therapeutic data from patient medical records. We used means (+SD) to describe continuous variables, and percentages and 95% CIs to describe categoric variables. We explored associated factors with sepsis. We used Mann–Whitney tests to compare continuous data of independent samples where appropriate. We used the Fisher test of homogeneity for categoric variables. We used an α level of 0.05 for statistical tests, for which we also calculated SDs and 95% CIs.

## Results

### Population and Temporality of Infections

Data from 67 patients diagnosed with *Vibrio* infection were available for the period 2001–2019 (Table 1). Most patients were men (81%), and the average age was 54 years (SD +24 years). In the subgroup of patients with acute infections (including cutaneous infections and gastroenteritis), the mean age was 60 (SD +20) years, and 71% of the patients had >1 underlying condition. Patients with chronic ear infections were younger (mean age 27 years [SD +24 years]), and all but 1 had no underlying conditions. 

**Table 1:** Clinical characteristics of patients with *Vibrio* infection, by species, Bay of Biscay, France, 2001–2019*

Characteristic	*V. alginolyticus*	*V. parahaemolyticus*	*V. cholerae* non-O1/O139	*V. vulnificus*	Other species
Total patients	23 (100)	20 (100)	10 (100)	7 (100)	7 (100)
Demographics					
Age, y, median (SD)	50 (+26.7)	53 (+22.8)	69 (+19.7)	66 (+11.5)	40 (+24.8)
Sex					
M	19 (83)	15 (75)	7 (70)	7 (100)	6 (86)
F	4 (17)	5 (25)	3 (30)	0	1 (14)
Underlying condition					
Heart failure	8 (35)	6 (30)	5 (50)	4 (57)	1 (14)
Neoplasia	1 (4)	5 (25)	4 (40)	0 (0)	1 (14)
Diabetes	2 (9)	3 (15)	1 (10)	1 (14)	1 (14)
Kidney failure	2 (9)	1 (5)	1 (10)	0	3 (43)
Immune disease	2 (9)	2 (10)	1 (10)	0	2 (29)
Hemopathy	1 (4)	1 (5)	1 (10)	1 (14)	1 (14)
Liver disease	1 (4)	1 (5)	2 (20)	1 (14)	0
Alcohol use disorder	2 (9)	1 (5)	2 (20)	2 (29)	0
Preexisting wound	3 (13)	0	0	3 (43)	0
Digestive surgery	2 (9)	2 (10)	1 (10)	0	1 (14)
Time to symptom onset, d, median (SD)	2.4 (+2.0)	1.3 (+0.9)	3 (+4.4)	5.6 (+8.1)	1 (+0.0)
Infection type					
Acute	14 (61)	19 (95)	10 (100)	7 (100)	5 (71)
Chronic	9 (39)	1 (5)	0	0	2 (29)
Outcome					
Recovered	21 (91)	17 (85)	8 (80)	6 (86)	7 (100)
Died	2 (9)	3 (15)	2 (20)	1 (14)	0

*Values are no. (%) except as indicated.

The description of environmental factors was available for 57% of patients. Among those patients, 55.3% of infections were contracted at the beach, 39.5% by handling or eating seafood, and 5.3% while abroad. Most infections (82%) occurred during June–September. The number of reported cases reached 2 peaks, in 2003 and 2018.

### Clinical Features

The average duration between known exposure and onset of symptoms was 2.4 days (SD +3.8 days), and it varied from <1 day for patients with gastroenteritis, cellulitis, or pneumonia, to 20 days for patients with osteitis. Digestive disorders were reported in 23 (34.4%) of the patients, including 6% with severe intraabdominal infection (Table 1). Cellulitis was reported in 23 (34.4%) of the patients, and 3 had soft tissue infection complicated by osteitis. Near drowning–associated pneumonia was reported in 8 (12%) of the patients. A case of endocarditis was described in a patient whose pacemaker had been exposed to seawater through a preexisting chronic wound while swimming in the Atlantic Ocean. Chronic ear infection (chronic otitis or cholesteatoma) affected 12 (18%) of the patients.

### Diagnostic Testing, *Vibrio* Species, and Drug-Susceptibility Testing

*Vibrio* infections were diagnosed from blood samples (26.9%), feces (20.9%), biopsies (20.9%), ear swab samples (17.9%), bronchoalveolar lavage samples (7.5%), and skin samples (6%). The most frequently identified species were *V. alginolyticus* (34%) and *V. parahaemolyticus* (30%). *V. cholerae* non-O1/O139 was found in 15% of patients, and *V. vulnificus* was found in 10%. The remaining patients were infected with other *Vibrio* species. Other bacteria were co-isolated in samples from 5 patients (methicillin-sensitive *Staphylococcus aureus* in 2 skin samples; *Streptococcus mitis* in a bronchoalveolar lavage; *Proteus vulgaris* and *Haemophilus influenza* in another bronchoalveolar lavage; and *Klebsiella pneumoniae*, *Enterococcus faecalis*, and *Enterobacter cloacae* in a bone biopsy).

Susceptibility testing revealed strains with resistance or intermediate resistance to amoxicillin in most *V. alginolyticus*, *V. parahaemolyticus*, and *V. cholerae* non-O1/O139 strains (Table 2). Strains with resistance or intermediate resistance to ticarcillin were also found in most *V. alginolyticus* and *V. parahaemolyticus* strains and to a lesser extent in *V. cholerae* non–O1/O139 strains. *V. vulnificus* strains were sensitive to all of these penicillins.

**Table 2:** Available drug-susceptibility test results for the main antibiotics used to treat *Vibrio* infections, by species, Bay of Biscay, France, 2001–2019*

Antibiotic	*V. alginolyticus*				*V. parahaemolyticus*				*V. cholerae* non-O1/O139				*V. vulnificus*		
	S	I	R		S	I	R		S	I	R		S	I	R
Amoxicillin	1	0	15		1	6	7		2	2	3		5	0	0
Ticarcillin	5	0	10		2	2	9		5	0	1		5	0	0
First-generation cephalosporin	10	4	0		13	1	0		4	1	0		4	1	0

*Data are no. of cases. I, intermediate; R, resistant; S, susceptible.

### Diseases Caused by *Vibrio* infection

*V. alginolyticus* was responsible for various pathologies, but more particularly for otitis (39%) (Figure). *V. parahaemolyticus* was identified in patients with cellulitis (40%) and gastroenteritis (40%). *V. cholerae* non-O1/O139 was almost exclusively responsible for digestive disorders (90%). *V. vulnificus* was exclusively found in cellulitis and soft tissue infections complicated by osteitis.

**Figure:**
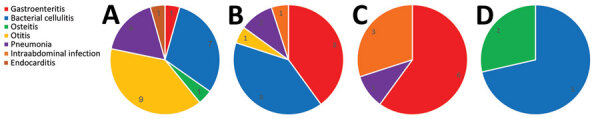
Diseases caused by *Vibrio* infection in 67 patients, by species, Bay of Biscay, France, 2001–2019. A) *V. alginolyticus*. B) *V. parahaemolyticus.* C) *V. cholerae* non-O1/O139. D) *V. vulnificus*. Numbers in chart sections indicate number of patients. Intraabdominal infection corresponds to pancreatitis, liver abscess, phlegmoneous ileitis, cholecystis, and peritonitis.

### Treatment

Most (84%) patients required hospitalization. The average time from symptom onset to treatment was 2.7 days (SD +4.9) days. Most of the patients received antibiotics (90%), of whom >50% received a multidrug regimen. The main prescribed antibiotics were penicillins (91%), quinolones (36%), cephalosporins (30%), metronidazole (15%), tetracycline (10%), and aminoglycosides (9%).

Twenty-two patients (33%) underwent surgery. Eleven patients with necrotizing cellulitis and 3 patients with osteitis required surgical debridement. For 6 of those 11 patients, amputation was necessary. Five patients with chronic ear infection required either surgical excision (n = 3), meatotomy (n = 1), or tympanoplasty (n = 1). Two patients had a cholecystectomy, and 1 patient with phlegmonous ileitis had partial colectomy.

### Factors Associated with Severe Forms

All patients with chronic infection were cured. Among patients with acute infection, 13 (24%) went into septic shock (Table 3), 6 (11%) had amputations, and 8 (14%) died. Half of the amputations were associated with *V. vulnificus* infections. Older age and malignant hemopathy (e.g., acute leukemia and lymphoma under chemotherapy) were associated with death. Three patients suffered pneumonia after near drowning, and death may have been attributable to cardiorespiratory arrest and intensive care complications. A probable link between *Vibrio* infection and death could be established for 5 patients. The case-fatality rate was the highest for *V. vulnificus* infections (1 attributable death out of 7 infections), followed by *V. parahaemolyticus* (2 attributable deaths out of 19 infections) and *V. cholerae* non-O1/O139 (1 attributable death out of 10 infections). The case-fatality rate was the lowest for *V. alginolyticus* infections (1 attributable death out of 14 infections).

**Table 3:** Clinical characteristics and outcome of patients with and without septic shock after acute *Vibrio* infection, Bay of Biscay, France, 2001–2019*

Characteristic		No sepsis, n = 42			Septic shock, n = 13		p value
		No.	% (95% CI)		No.	% (95% CI)	
Patient sex							
M		35	83 (72–95)		10	77 (54–100)	0.685
F		7	17 (5–28)		3	23 (0.2–46)	
Underlying conditions							
Heart failure		18	43 (28–58)		6	46 (19–73)	Referent
Neoplasia		6	14 (4–25)		4	31 (6–56)	0.223
Diabetes		7	17 (5–28)		1	8 (0–22)	0.664
Kidney failure		5	12 (2–22)		2	15 (0–35)	0.664
Immune disease		5	12 (2–22)		2	15 (0–35)	0.664
Hemopathy		3	7 (0–15)		2	15 (0–35)	0.582
Liver disease		2	5 (0–11)		3	23 (0–46)	0.318
Alcohol use disorder		3	7 (0–15)		4	31 (6–56)	0.102
Preexisting wound		6	14 (4–25)		0	0 (0–0)	0.317
Digestive surgery		4	10 (1–18)		2	15 (0–35)	0.618
Species							
* V. alginolyticus*		10	24 (11–37)		4	31 (6–56)	
* V. parahaemolyticus*		14	33 (19–48)		5	38 (12–65)	
* V. cholerae* non-O1/O139		8	19 (7–31)		2	15 (0–35)	
* V. vulnificus*		6	14 (4–25)		1	8 (0–22)	
Other *Vibrio* species		4	10 (1–18)		1	8 (0–22)	
Outcome							
Recovered		40	95 (89–100)		7	54 (27–81)	0.001
Died		2	5 (0–11)		6	46 (19–73)	

*Median patient age (+SD) was 60 (+21.4) for no sepsis and 61 (+15.3) for septic shock.

## Discussion

The cases of *Vibrio* infections reported in this study are the most severe cases that ended up requiring hospitalization. Non–*V.* *cholerae* and *V. cholerae* non-O1/O139 bacteria can cause mild diarrhea and gastroenteritis, for which patients typically are not hospitalized (*7*,*14*), and the number of vibriosis incidents per year in the region is probably higher that those reported in our study. Comparing the demographics of our population with those described in a 1996–2010 review surveillance in the United States (*10*), we observed a higher proportion of men (81% vs. 68%), and the age group with the highest percentage of cases was 60–69 years in our population compared with 40–49 years in the United States. This difference is probably attributable to the fact that our population mainly consists of the most severe cases of infection that occur most often in older person (*15*). *Vibrio* infections are usually initiated from exposure to contaminated water or consumption of raw or undercooked contaminated seafood. As reported in 2008 by Dechet et al. (*16*), seawater-related activities as simple as walking on the beach can lead to *Vibrio* infections (*16*), which was also reported for >50% of the patients with environmental factors identified in our study. *Vibrio* species are responsible for 20% of bacterial illnesses related to shellfish consumption (*17*). In our study, 39% of the cases were acquired after seafood handling or consumption. *Vibrio* bacteria caused more seafood-associated outbreaks during the warmer months (*11*), and all but 1 case occurred during June–September in our study population. Extreme heat waves led to unprecedented high sea surface temperatures, which appear to be responsible for the emergence of *Vibrio* bacteria in areas where they are usually not present (*18*,*19*). In 2003, France experienced the hottest summer in a century, which may have led to an increase in the concentration of *Vibrio* on the Bay of Biscay, given that the number of reported *Vibrio* infections also increased this year.

We compared the results of our study to a similar investigation conducted in the US state of Florida (*13*). The most common species reported in Florida over 10 years were *V. vulnificus* (33.1%), *V. parahaemolyticus* (29.4%), *V. alginolyticus* (15.7%), and *V. cholerae* non-O1/O139 (6.6%). In our study, we report a slightly different distribution: *V. alginolyticus* (34.3%), *V. parahaemolyticus* (29.9%), *V. cholerae* non-O1/O139 (14.9%), and *V. vulnificus* (10.4%)*.* The incubation period of *V. parahaemolyticus*, *V. vulnificus* (when exposed to a wound), and *V. cholerae* non-O1/O139is <24 hours; for other clinical manifestations after infection with *V. vulnificus*, the incubation period ≈48 hours (*7*,*14*). In our study, the time between known exposure and onset of symptoms was <48 hours in 74% of cases.

Clinical manifestations are different depending on the type of *Vibrio* species. *V. alginolyticus* has been identified as a relevant cause of superficial wound and ear infections (*14*). In our study, *V. alginolyticus* was responsible for most cases of chronic otitis. However, contrary to what has been observed in Florida (*13*), this species also caused 1 death associated with wound infection. *V. parahaemolyticus* is the most prevalent foodborne bacterium associated with seafood consumption and typically causes acute gastroenteritis (*20*), but it has also been identified in wound-associated cases (*13*). In our study, most *V. parahaemolyticus* infections caused either gastroenteritis or bacterial cellulitis, but the species was also responsible for pneumonia, phlegmonous ileitis, and otitis. *V. cholerae* non-O1/O139 is the causative agent of gastrointestinal and extraintestinal infections and has been reported to be the cause of one third of deaths in infected patients (*21*). Of the 10 patients with *V. cholerae* non-O1/O139 infection reported in this study, 6 had >1 risk factor (e.g., cancer or malignant blood diseases, alcoholism, other liver diseases, and diabetes), and 1 died from the infection. *V. vulnificus* infections in Europe are rare and sporadic (*22*) but have the highest reported case-fatality rate of any foodborne pathogen (*12*,*23*). In our study, 7 total cases were reported in 2005, 2007, 2015, 2017, and 2018, and 4 resulted in either amputation, septic shock, or death.

Because *Vibrio* infections can cause severe reaction or disease, treatment with a combination of a third-generation cephalosporin and a tetracycline or a fluoroquinolone alone is recommended. Higher mortality rates were observed with a β-lactam alone, compared with fluoroquinolone alone or fluoroquinolone or tetracycline plus a β-lactam (*24*). In the United States, the most commonly used antibiotics for patients with *Vibrio* infections were quinolones (56.1%), followed by cephalosporins (24.1%), tetracyclines (23.5%), and penicillins (15.4%) (*24*). Less than one third of patients with *Vibrio* infections received appropriate antibiotic therapy (*13*). According to our study, in France, the main prescribed antibiotics for *Vibrio* infections were penicillins (91%), quinolones (36%), cephalosporins (30%), metronidazole (15%), and tetracycline (10%), and >50% of patients received a multidrug regimen.

The main limitations of our study are that vibriosis is not a notifiable disease in France, that not all hospitals in the Bay of Biscay participated, and that the reported cases probably underestimated the situation. Data were also not always complete on each case-patient, and details of food histories or other exposures were not always available.

In conclusion, the incidence of serious marine-related *Vibrio* infections has been low on the west coast of France. However, predicted rising ocean temperatures and demographic shifts (e.g., an aging population with increased risk factors) may lead to the emergence of opportunistic vibriosis in France and other coastal countries in temperate and tropical regions. Our retrospective case-series study provides a basis for identifying and treating new cases of *Vibrio* infections that might affect larger population sectors in the future.
